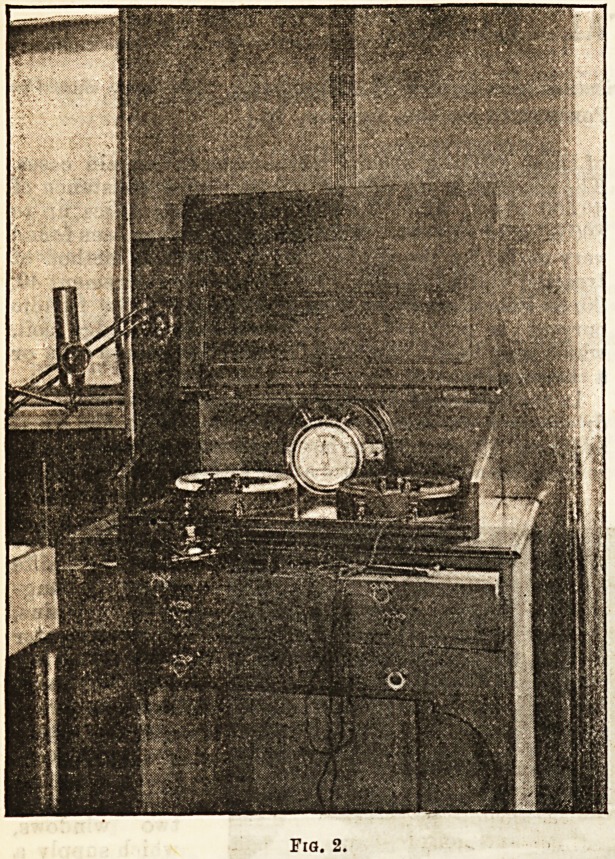# Arrangement for Electrical Treatment

**Published:** 1893-07-08

**Authors:** 


### THE HOSPITAL CLINIC.

[*The Editor will be glad to receive offers of co-operation and contributions from members of the profession. All letters should be addressed to* THE EDITOR, THE LODGE, PORCHESTER SQUARE, LONDON, W.]

### GLASGOW WESTERN INFIRMARY.

### ARRANGEMENT FOR ELECTRICAL TREATMENT.

On visiting this hospital we were pleased to note the very extensive arrangements for the electrical treatment of medical and surgical cases. The installation is one of the most complete we have seen in any of the hospitals we have visited, and embraces battery and engine room for the generation and storage of electricity, a completely fitted up electrical department with an attached bath-room for the treatment of out-door patients; these are all conveniently situated in the basement flat adjacent to the patients' entrance, and under the charge of a specially - trained sister.

The electrical treatment of in-patients is also extensively provided for, wires being carried from the battery room to each ward; a trolly carrying all the apparatus necessary for the application of faradic and galvanic currents forms part of the furnishing of each set of wards, and can be attached to terminals in the wall conveniently situated, and from which the current is derived. The general operating theatre and operating rooms are likewise provided with all the necessary apparatus for electrical treatment, both medical and surgical. A special feature in this department is the arrangement of the faradic and continuous currents for the treatment of chloroform accidents, the mere turning of a swi ch being sufficient to supply the necessary current at once. A reserve of electricity for use in cases of surgical emergency is provided by a trolly carrying secondary cells, which supply sufficient current for cautery and endoscopic work. A short description of the furnishings of the various departments may prove of interest to our readers.

The battery room is fitted with shelves upon which are arranged 60 cell Leclanche batteries for supplying the galvanic current, the total number of cells in use being over 400. These have been found most economical and require comparatively little attention. The storage cells for surgical use are arranged in the same room, and are charged by a dynamo driven by a three-horse power Otto gas engine, which also supplies electric light for the operating theatre. The electrical room is well lit and suitably arranged; it contains on one side a complete set of apparatus for the treatment of medical patients. The continuous current is so arranged that it can be used either directly or in dividedcircuit, each of these offering special advantages in certain cases. The rheostat is capable of interposing a resistance of 40,000 ohms, while the galvanometre measures up to 800 m. a. of current. This arrangement has been found very useful for the application of Apostolis method of treating uterine tumors. By a switch arrangement 40, 60, or 100 cells may be used as required. The faradic current is provided by Lewandowsky's sledge coil, which may be set in motion by four Leclanche cells or a thermopyle.

In the centre of the room is a large static machine, driven by a water motor, and provided with the usual adjuncts. This apparatus has been found specially useful in chorea and in neuralgic and hysterical affections.

FIG. 1.

On the table supporting the static machine is fixed a Gaiffe electro - magnetic machine, driven by water power. This has sometimes been found to prove useful where the faradic current is inapplicable. Between two windows, which supply a brilliant light, is a stand supporting two large rheostats, one of high and the other of low resistance, used for lamp and cautery work. The lamps are used with the cystoscope, and other forms of endoscope, for the examination of the stomach, uterus, rectum, and other cavities.

The bath-room adjoins the electrical room, and is arranged for the application of faradic and galvanic baths. This is a neat little room with a fireplace, and is made as comfortable as possible consistent with the use to which it is put.

The general operating theatre is very completely fitted up. On one side is a table fitted with rheostats and ampere meter, while on the other side is a similar table fitted up wi'h rheostats, switch, and current reverser for the continuous current, and a De Watteville's coil and a De Watteville's primary coil. From these wires are led to a central switchboard, conveniently placed to the operating table. This switchboard is arranged so that the current may be taken for electrolysis, faradaism, cautery, light, or for driving a small motor, which can be used for trephining or drilling, &c.

The theatre is lit by electricity, but in addition a lantern with bull's eye arrangement, and carrying a sixteen-candle lamp can be used for throwing a brilliant light over a limited area, and has been found very serviceable in cerebral and abdominal operations.

The accompanying cuts are taken from photographs, and explain themselves.

Fig. 2.

Owing to the extensive use of electricity in this hospital the nurses receive special instructions in the manipulation of the various apparatus already described, and require to show proficiency in this special branch of medical and surgical treatment before receiving their certificates.

## Figures and Tables

**Fig. 1. f1:**
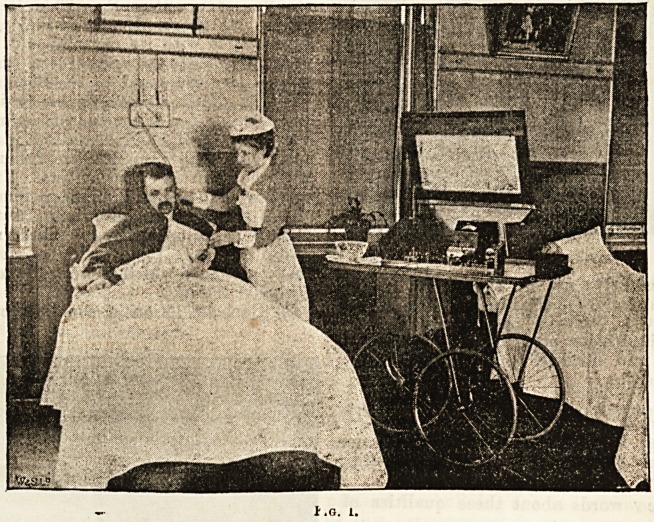


**Fig. 2. f2:**